# The BTG4 and CAF1 complex prevents the spontaneous activation of eggs by deadenylating maternal mRNAs

**DOI:** 10.1098/rsob.160184

**Published:** 2016-09-07

**Authors:** Michał Pasternak, Sybille Pfender, Balaji Santhanam, Melina Schuh

**Affiliations:** 1 Medical Research Council, Laboratory of Molecular Biology, Francis Crick Avenue, Cambridge Biomedical Campus, Cambridge CB2 0QH, UK; 2 Max Planck Institute for Biophysical Chemistry, Am Fassberg 11, 37077 Göttingen, Germany

**Keywords:** oocyte, meiosis, cell cycle, deadenylation

## Abstract

Once every menstrual cycle, eggs are ovulated into the oviduct where they await fertilization. The ovulated eggs are arrested in metaphase of the second meiotic division, and only complete meiosis upon fertilization. It is crucial that the maintenance of metaphase arrest is tightly controlled, because the spontaneous activation of the egg would preclude the development of a viable embryo (Zhang *et al.* 2015 *J. Genet. Genomics* **42**, 477–485. (doi:10.1016/j.jgg.2015.07.004); Combelles *et al.* 2011 *Hum. Reprod.* **26**, 545–552. (doi:10.1093/humrep/deq363); Escrich *et al.* 2011 *J. Assist. Reprod. Genet.* **28**, 111–117. (doi:10.1007/s10815-010-9493-5)). However, the mechanisms that control the meiotic arrest in mammalian eggs are only poorly understood. Here, we report that a complex of BTG4 and CAF1 safeguards metaphase II arrest in mammalian eggs by deadenylating maternal mRNAs. As a follow-up of our recent high content RNAi screen for meiotic genes (Pfender *et al.* 2015 *Nature* **524**, 239–242. (doi:10.1038/nature14568)), we identified *Btg4* as an essential regulator of metaphase II arrest. *Btg4-*depleted eggs progress into anaphase II spontaneously before fertilization. BTG4 prevents the progression into anaphase by ensuring that the anaphase-promoting complex/cyclosome (APC/C) is completely inhibited during the arrest. The inhibition of the APC/C relies on EMI2 (Tang *et al.* 2010 *Mol. Biol. Cell* **21**, 2589–2597. (doi:10.1091/mbc.E09-08-0708); Ohe *et al.* 2010 *Mol. Biol. Cell* **21**, 905–913. (doi:10.1091/mbc.E09-11-0974)), whose expression is perturbed in the absence of BTG4. BTG4 controls protein expression during metaphase II arrest by forming a complex with the CAF1 deadenylase and we hypothesize that this complex is recruited to the mRNA via interactions between BTG4 and poly(A)-binding proteins. The BTG4–CAF1 complex drives the shortening of the poly(A) tails of a large number of transcripts at the MI–MII transition, and this wave of deadenylation is essential for the arrest in metaphase II. These findings establish a BTG4-dependent pathway for controlling poly(A) tail length during meiosis and identify an unexpected role for mRNA deadenylation in preventing the spontaneous activation of eggs.

## 1. *Btg4*-depleted eggs progress spontaneously into anaphase II

**Figure 1.**
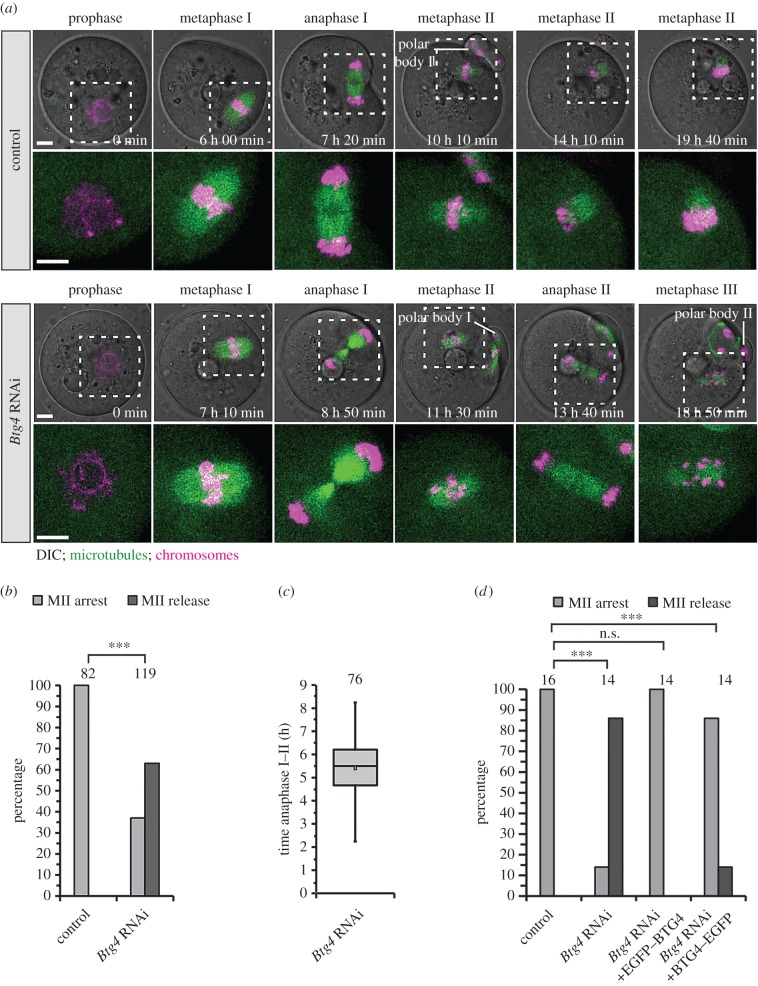
Depletion of *Btg4* triggers spontaneous resumption of meiosis. (*a*) Representative examples of oocytes microinjected with siRNAs targeting *Btg4* or injected with control siRNA. Region of spindle and chromosomes, highlighted in the top panel, is magnified without DIC below. Scale bars, 10 µm. Time stamp denotes time after the start of imaging. (*b*) Sixty-three percent of *Btg4*-depleted oocytes spontaneously resumed meiosis and progressed into anaphase II, whereas 100% of control oocytes remained arrested at metaphase II. (*c*) Time between the onset of anaphase I and anaphase II in *Btg4-*depleted oocytes. (*d*) Rescue experiments: microinjection of mRNA encoding *EGFP–Btg4* into *Btg4*-depleted (the same mix of siRNAs as in *a*–*c*) oocytes prevented spontaneous resumption of meiosis. The number of analysed oocytes is specified above the bars. *p*-Values were calculated with Fisher's exact test and *** denotes *p*-value < 0.0001. Data from 12 experiments for (*b*), 10 for (*c*) and two for (*d*). See also electronic supplementary material, figure S1 and movie S1.

We recently carried out a high content RNAi screen to identify new meiotic genes in mammalian oocytes [1]. In the screen, pools of 12 genes were targeted simultaneously by microinjecting mixes of siRNAs. Many of the siRNA mixes caused defects during oocyte meiosis, but for several mixes, the identity of the genes that caused the phenotypes remained to be determined. We focused on a mix that caused eggs to progress into anaphase II upon forming the second metaphase spindle. Stepwise splitting of the siRNA mix identified *Btg4* as the gene that caused the release. *Btg4*-depleted oocytes had no apparent defects during the first meiotic division (figure 1*a*): they underwent nuclear envelope breakdown (NEBD) and anaphase I with similar efficiency and timing as oocytes microinjected with control siRNAs (electronic supplementary material, figure S1*a*–*d*). In addition, the formation of metaphase II (MII) spindles was not affected (figure 1*a*). However, 63% of *Btg4*-depleted oocytes spontaneously progressed into anaphase II within 5.4 ± 1.7 h after anaphase I, whereas the control oocytes remained arrested in metaphase (figure 1*a*–*c* and electronic supplementary material, movie S1). This precocious release from metaphase II arrest was accompanied by a high incidence of lagging chromosomes (electronic supplementary material, figure S1*e*). Under physiological conditions, the release from metaphase II arrest is triggered by fertilization and results in meiotic exit and the formation of a pronucleus [2–6]. The *Btg4*-depleted oocytes that underwent anaphase II proceeded directly into a non-physiological third meiotic division (MIII) and formed a distorted MIII spindle that fragmented and had misaligned chromosomes (figure 1*a*). This phenotype was specifically due to the depletion of *Btg4*, because it could be rescued by the expression of EGFP-tagged BTG4 (figure 1*d*).

## 2. BTG4 is required to inhibit the anaphase-promoting complex/cyclosome during metaphase II arrest

**Figure 2.**
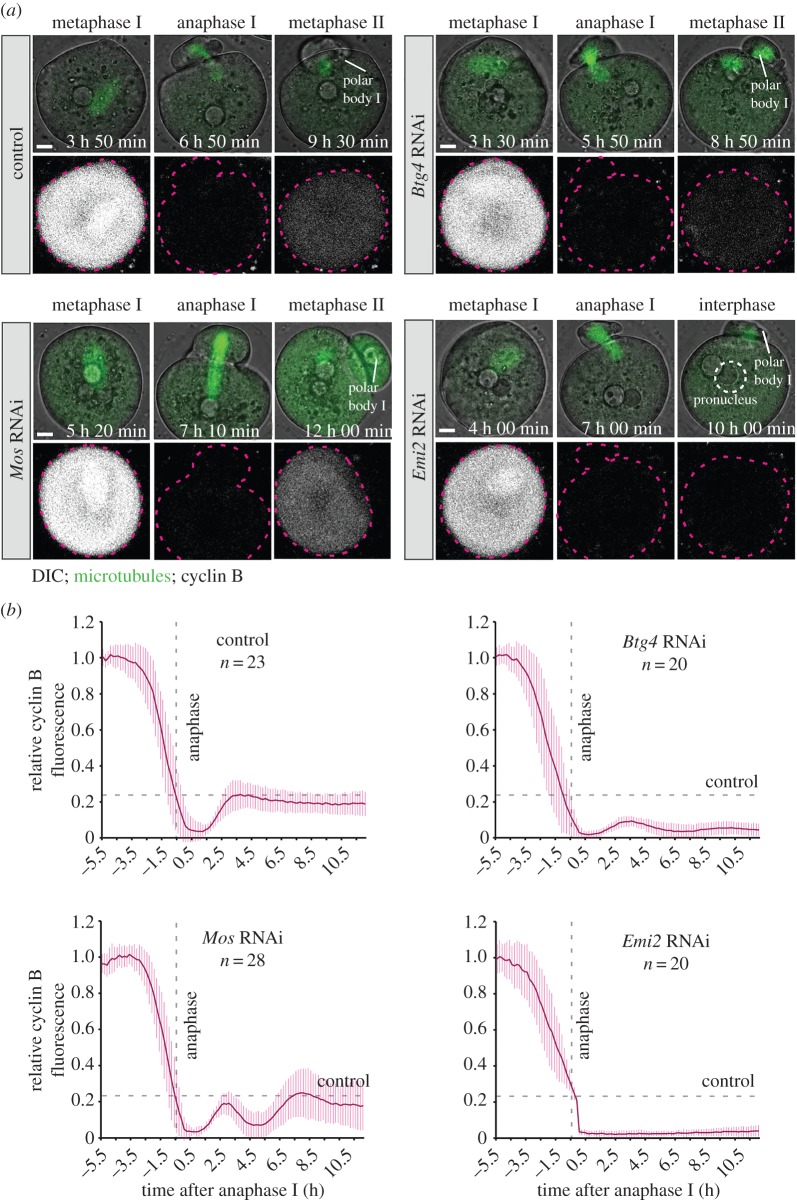
*Btg4*-depleted oocytes fail to inhibit the APC/C. (*a*) Representative examples of oocytes microinjected with siRNAs targeting *Btg4, Mos, Emi2* or microinjected with control siRNA and expressing fluorescent cyclin B during oocyte maturation. Scale bars, 10 µm. Time stamp denotes time after the start of imaging. Pink dashed line outlines the oocyte. (*b*), Quantifications of cyclin B signal intensity measured in the middle section of each oocyte, reflecting changes in APC/C activity over time (time 0 was set as anaphase onset). High fluorescence correlates with low APC/C activity. Vertical dashed lines indicate anaphase onset and horizontal dashed lines indicate the fluorescence recovery in control oocytes during metaphase II. Data from two independent repetitions. See also electronic supplementary material, figure S2 and S3.

The maintenance of metaphase II arrest crucially relies on the inhibition of the anaphase-promoting complex/cyclosome (APC/C). The APC/C is activated at the metaphase-to-anaphase transition during the first meiotic division, when it triggers the progression into anaphase by targeting cyclin B and securin for degradation. Subsequently, it becomes inactivated and inhibited during metaphase II arrest, so that the levels of cyclin B and securin remain high [7–10]. We tested if the spontaneous release from metaphase II arrest in *Btg4*-depleted oocytes was due to impaired inhibition of the APC/C by monitoring the levels of APC/C activity in live oocytes throughout maturation. To this end, we expressed fluorescent cyclin B and securin in oocytes undergoing meiosis and quantified their intensity. As expected, cyclin B and securin levels were high after NEBD and both proteins were rapidly degraded before the onset of anaphase I, when the APC/C was activated (figure 2 and electronic supplementary material, figure S2*a*). In control oocytes, the levels of cyclin B and securin were partially restored after the extrusion of the first polar body, when the metaphase II spindle was formed (figure 2 and electronic supplementary material, figure S2*a*), indicative of effective inhibition of the APC/C. In contrast, *Btg4*-depleted oocytes failed to re-accumulate securin and cyclin B after the completion of the first meiotic division, indicating that the APC/C was not efficiently inhibited. Residual inhibition of the APC/C was probably achieved, because the levels of cyclin B and securin still slowly increased following anaphase I, albeit at a much lower rate than in control oocytes. The cyclin B and securin levels decreased again when the oocytes progressed into anaphase II (figure 2).

Two major pathways are known to mediate metaphase II arrest and the inhibition of the APC/C [6,11]. The first pathway relies on EMI2, a direct inhibitor of the APC/C [12].

EMI2 is expressed only when the oocytes have progressed into anaphase I and is required for both the establishment and the maintenance of metaphase II arrest, and *Emi2*-depleted oocytes enter interphase and form a pronucleus after completion of the first meiotic division [13,14] (electronic supplementary material, figure S3*a*,*e*). The second pathway involves the MOS/MEK/MAPK signalling cascade and is required for the maintenance of metaphase II arrest [15,16]. *Mos*-depleted oocytes undergo two consecutive meiotic divisions and proceed to metaphase III, similarly to *Btg4*-depleted oocytes (electronic supplementary material, figure S3*a*–*c*). However, in contrast to *Btg4*-depleted oocytes, they extrude abnormally large polar bodies and transiently form a nucleus before entering second meiotic division (electronic supplementary material, figure S3*a*–*c*). The exact mechanism by which the MOS/MEK/MAPK pathway mediates metaphase II arrest in mammalian oocytes remains to be determined. Given the essential function of the EMI2- and MOS-dependent pathways in metaphase II arrest, we tested if the activity of these pathways is affected upon *Btg4* depletion.

First, we compared the degree of APC/C inhibition in *Btg4*-, *Mos-* and *Emi2-* depleted oocytes by analysing the levels of cyclin B and securin in real time, using live fluorescence microscopy. In contrast to *Btg4*-depleted oocytes, *Mos*-depleted oocytes were able to inhibit the APC/C after the completion of the first meiotic division, as indicated by the re-accumulation of cyclin B and securin (figure 2 and electronic supplementary material, figure S2*a*). In contrast, *Emi2*-depleted oocytes were unable to inhibit the APC/C as evident from the complete degradation of cyclin B and securin (figure 2 and electronic supplementary material, figure S2*a*). The inhibition of the APC/C was thus severely impaired in both *Emi2*- and *Btg4*-depleted oocytes, with slightly lower levels of APC/C activity in *Btg4*-depleted oocytes.

We then tested directly if either the MOS/MEK/MAPK or EMI2 pathways were affected upon *Btg4* depletion. RNAi against *Btg4* did not perturb MAPK activation during metaphase II as evident from the level of phosphorylated and thus active MAPK (electronic supplementary material, figure S2*b*). In contrast, *Btg4* depletion did indeed impair the EMI2-dependent pathway: western blots with an EMI2-specific antibody revealed that the levels of EMI2 were strongly decreased upon *Btg4* depletion (electronic supplementary material, figure S2*b*). The impaired inhibition of the APC/C in the absence of BTG4 as well as the release from metaphase II arrest could thus be facilitated by the decrease in EMI2 levels.

## 3. BTG4 forms a functional complex with CAF1 deadenylase

**Figure 3.**
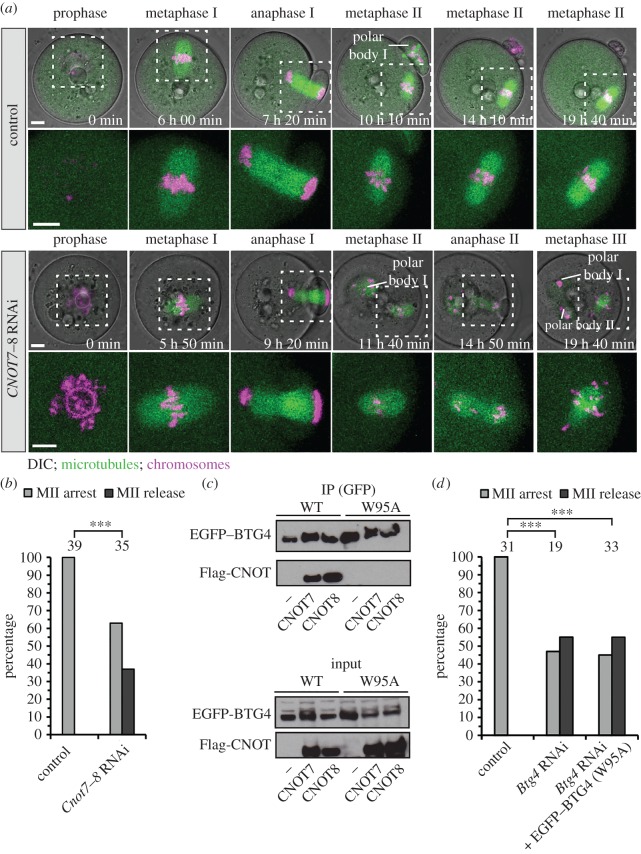
BTG4 forms a functional complex with CAF1 (CNOT7 and CNOT8) deadenylase. (*a*) Representative examples of oocytes microinjected with siRNAs targeting the *Cnot7* and *Cnot8* subunits of CCR4–NOT deadenylase or control siRNA. Region of spindle and chromosomes, highlighted in the upper panel, is magnified without DIC below. Scale bars, 10 µm. Time stamp denotes time after the start of imaging. (*b*) Thirty-seven percent of *Cnot7–8*-co-depleted oocytes spontaneously resumed meiosis and progressed through anaphase II. (*c*) Co-immunoprecipitation and western blot on HeLa cells expressing wild-type EGFP–BTG4 (WT) or mutant (W95A) together with Flag-CNOT7 or Flag-CNOT8. Only wild-type BTG4 interacted with CNOT7 and CNOT8. (*d*) Rescue experiments: injection of mRNA encoding mutant EGFP–BTG4 (W95A) into *Btg4*-depleted oocytes did not rescue the phenotype. The number of analysed oocytes is specified above the bars. *p*-Values were calculated with Fisher's exact test and *** denotes *p*-value < 0.0001. Data from six experiments for (*b*), and three for (*c*) and (*d*). See also electronic supplementary material, figure S4 and movie S2.

The decrease in EMI2 levels upon BTG4 depletion suggested a function of BTG4 in controlling gene expression. Consistent with such a function, exogenous EGFP-tagged BTG4 localized to both the nucleus and the cytoplasm of a maturing oocyte, where it could be involved in controlling gene expression (electronic supplementary material, figure S1*d*). A recent study reported a similar pattern of cellular localization, yet showed that the endogenous protein is synthesized only in metaphase II [17]. To identify the mechanism by which BTG4 affects protein expression, we searched systematically for interaction partners of BTG4 by performing a yeast two-hybrid screen (Y2H). The screen identified several potential interaction partners, including CNOT7 and CNOT8 (electronic supplementary material, table S1). These proteins are homologues of CAF1, a catalytic subunit of the CCR4–NOT deadenylase complex [18,19]. CCR4–NOT is a major cellular deadenylase, which shortens the poly(A) tails of mRNAs [20]. The deadenylation leads to translational silencing of the target mRNAs and typically initiates the cytoplasmic decay of the respective mRNA [21,22]. Given that CNOT7 and CNOT8 can function redundantly [23], we co-depleted both of them. Co-depletion resulted in a release from metaphase II arrest, mirroring the phenotype of *Btg4* depletion (figure 3*a*,*b*; electronic supplementary material, figure S4*a* and movie S2). In addition, co-depletion of *Cnot7* and *Cnot8* caused chromosome alignment defects, lagging chromosomes during anaphase I and an increase in the fraction of oocytes that arrested in metaphase I (electronic supplementary material, figure S4*b*–*c*). This wide range of defects is likely to reflect the importance of the CCR4–NOT complex in several steps of meiosis and indicates that it has a broad spectrum of targets [18]. To test if BTG4 and CNOT7 or CNOT8 interact directly, we mutated a predicted interaction site within the BTG4 APRO domain, based on homology with BTG2 [24]. The introduced mutation (W95A) abolished the interaction with CNOT7 and CNOT8 (figure 3*c*). Importantly, the mutant BTG4 was unable to rescue metaphase II arrest in *Btg4*-depleted oocytes (figure 3*d*). This shows that BTG4's function during oocyte maturation crucially depends on its interaction with CNOT7 and CNOT8, and suggests that BTG4 prevents the spontaneous resumption of meiosis by controlling mRNA deadenylation.

BTG4 does not contain any known RNA-binding domain. It is thus likely to rely on a second interaction partner to mediate binding of the CCR4–NOT complex to mRNA. A recent study suggested that BTG4 may interact with eIF4E to bind to mRNAs [17]. Interestingly, our Y2H screen identified PABPN1 as additional binding partner of BTG4. PABPN1 binds directly to poly(A) tails and facilitates mRNA deadenylation by CCR4–NOT [25]. PAPBN1 could therefore be involved in mediating the recruitment of the BTG4–CCR4–NOT complex to target mRNAs. We depleted *Pabpn1* by RNAi and compared the resulting phenotypes with those caused by depleting *Btg4* or *Cnot7/Cnot8*. Microinjection of siRNAs targeting *Pabpn1* resulted in a release from metaphase II arrest in a subset of injected oocytes (electronic supplementary material, figure S4*f*) or caused a metaphase I arrest (electronic supplementary material, figure S4*g*), reminiscent of the phenotypes observed upon *Btg4* or *Cnot7/Cnot8* depletion. The model that poly(A)-binding proteins such as PABPN1 recruit the BTG4/CCR4–NOT complex to its target mRNAs is further supported by the fact that other members of the BTG/TOB family also interact with poly(A)-binding proteins [26]. Recently, BTG2 was shown to bridge CCR4–NOT to another poly(A)-binding protein, PABPC1 [27]. This suggests that the mechanism of the recruitment of BTG/TOB proteins to target mRNAs via poly(A)-binding proteins is likely to be conserved. However, the interaction between BTG4 and poly(A)-binding proteins remains to be confirmed *in vivo*.

## 4. Depletion of *Btg4* perturbs mRNA deadenylation and protein synthesis

**Figure 4.**
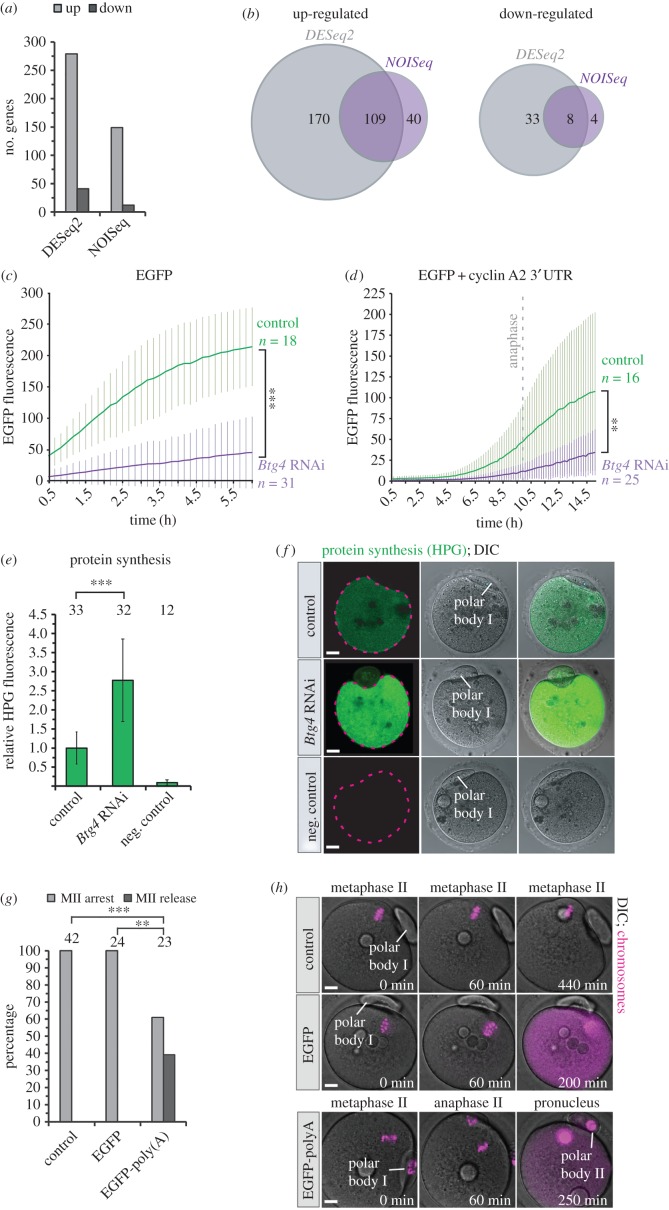
BTG4 limits global protein expression to enable the expression of proteins required for MII arrest. (*a*) Differentially-regulated genes upon *Btg4* depletion as indicated by DESeq2 or NOISeq analysis of RNA-seq results, showing an increase in the number of polyadenylated mRNAs. (*b*) Overlaps between DESeq2 and NOISeq analysis. (*c*) Expression of EGFP upon injection of mRNA into MII oocytes, showing significantly reduced levels of translation in *Btg4*-depleted oocytes. (*d*) Expression of EGFP upon microinjection of mRNA containing the 3′UTR of cyclin A2 into prophase-arrested oocytes, showing significantly reduced levels of translation in *Btg4*-depleted oocytes after the meiosis I–II transition. (*e*) Quantification of nascent protein synthesis during metaphase II in control and *Btg4*-depleted oocytes incorporating HPG. Representative examples are shown in (*f*). (*g*) Microinjection of polyadenylated mRNA encoding EGFP into metaphase II oocytes resulted in the spontaneous resumption of meiosis, while injection of a non-polyadenylated version had no effect. Representative examples are shown in (*h*). The increasing signal in the red channel (magenta) comes from bleed-through from the very strong EGFP signal at later time points after mRNA injection. Scale bars, 10 µm. The number of analysed oocytes is specified above the bars. Time stamps in (*c*,*d*,*h*) denote time after the start of imaging. *p*-Values were calculated with Fisher's exact test (*e*) or Student's *t*-test. For (*c*) and (*d*), the *t*-test was performed on the measurements from the final time points. *** denotes *p*-value < 0.0001, ** denotes *p*-value < 0.01. Data from three biological repetitions in (*a*) and (*b*), two experimental repetitions in (*c*,*d*,*e,f*) and three repetitions in (*g*) and (*h*). See also electronic supplementary material, movie S3.

To test for a role of BTG4 in mRNA deadenylation, we constructed cDNA libraries from mRNAs containing poly(A) tails in control and *Btg4*-depleted early MII oocytes (before the release from MII arrest) and compared the transcriptomes by RNA sequencing (RNA-seq). This revealed prominent differences in transcript abundancies in the poly(A)-enriched fraction. In particular, a large number of transcripts were upregulated in *Btg4*-depleted oocytes (figure 4*a*,*b* and electronic supplementary material, table S2). The increase in the fraction of poly(A) tailed mRNAs must have resulted from impaired mRNA deadenylation or degradation, because oocytes at this developmental stage do not synthesize new RNA [28,29]. Consistent with our findings, a recent study that was published while this manuscript was in preparation reported impaired mRNA deadenylation at the meiosis I–II transition in oocytes from *Btg4*^−/−^ mice [17]. The study showed that in oocytes from *Btg4^−/−^* mice, the timely degradation of mRNA at MI–MII transition is perturbed and that several tested mRNAs that should have been deadenylated at this transition, such as *Padi6, Zp2* and *Fgf2*, retained their poly(A) tails. However, what is the link between impaired mRNA deadenylation in *Btg4*-depleted oocytes and the release from metaphase II arrest? We observed that the levels of EMI2 were decreased upon *Btg4* depletion. Interestingly, the reduction of EMI2 was not due to a decrease in the amount of polyadenylated *Emi2* mRNA (electronic supplementary material, figure S2*c*). We hypothesized that the observed phenotypes could instead be caused by the presence of excess amounts of polyadenylated mRNA in the absence of BTG4. Transcripts that should normally be deadenylated or degraded during the MI–MII transition may occupy the translational machinery, for instance by sequestering poly(A) binding proteins. As a result, this could lead to an insufficient capacity of the oocyte to translate the mRNAs that are essential for metaphase II arrest, such as mRNAs encoding EMI2. To test this hypothesis, we microinjected identical amounts of mRNA encoding EGFP into control and *Btg4*-depleted oocytes in metaphase II. We observed that the expression of EGFP was decreased by 79% in *Btg4*-depleted oocytes (figure 4*c*). We also microinjected oocytes in prophase with mRNA encoding EGFP under the control of the 3′UTR of *cyclin A2*, which is known to be partly degraded during meiosis I and re-synthesized during metaphase II arrest [30]. The 3′UTR dictates when the mRNA is translated during the meiotic cell cycle [22,31,32]. As expected, expression of EGFP was low during meiosis I and increased at the MI–MII transition (figure 4*d*). We observed that the expression was decreased in *Btg4*-depleted oocytes by about 55% in comparison with the control group (figure 4*d*). To test if this reduction results from saturation of the translational machinery or overall inhibition of translation, we compared the translation rates in control and *Btg4*-depleted MII oocytes. To this end, we incubated oocytes that had completed the first meiotic division in medium containing l-homopropargylglycine (HPG), an analogue of methionine that can be detected using Click-IT chemistry (Life Technologies). The HPG signal that is indicative of the overall level of translation was nearly three times higher in *Btg4*-depleted oocytes than in controls (figure 4*e*,*f*), suggesting that global translation is increased upon Btg4 depletion. In further support of our hypothesis that the abundancy of polyadenylated mRNAs is responsible for the phenotype of *Btg4* depletion, microinjection of *in vitro* polyadenylated *EGFP* mRNA into metaphase II arrested oocytes triggered the onset of anaphase II, whereas microinjection of *EGFP* mRNA without the poly(A) tail had no effect (figure 4*g*,*h* and electronic supplementary material, movie S3). Collectively, these data show that *Btg4* depletion causes an increase in polyadenylated mRNAs and reduced expression of proteins that are normally expressed during metaphase II arrest. An increase in the abundancy of polyadenylated mRNAs and the resulting imbalance in protein expression are sufficient to trigger the spontaneous resumption of meiosis.

## 5. Discussion

Our *in vivo* analysis of the function of BTG4 sheds light on general mechanisms underlying metaphase II arrest in mammalian oocytes, a stage of critical importance for fertilization and the development of a viable embryo [6,11]. The detection of this phenotype required high-resolution imaging of live Btg4-depleted oocytes, which may explain why this phenotype was not reported in a recent study [17]. We propose that a complex of BTG4 and CCR4–NOT deadenylase is crucial for deadenylating a large number of mRNAs during metaphase II arrest (electronic supplementary material, figure S5). The large-scale deadenylation and degradation of mRNAs at the MI–MII transition has been well documented [33,34], yet its role remained elusive. Our data suggest that this large-scale deadenylation relieves pressure from the translational machinery and is a prerequisite for timely expression of several proteins that are essential later in meiosis for the maintenance of metaphase II arrest. This mechanism might ensure that the translational machinery is recruited to late instead of early meiotic transcripts. Our findings also highlight that experimentalists should avoid expressing high amounts of exogenous polyadenylated mRNAs in oocytes and embryos. As shown in this study, this can lead to an imbalance in the translation of endogenous proteins and cause non-specific defects.

The poly- and deadenylation of mRNAs is a major mechanism of regulating gene expression in oocytes [22,31], yet the proteins that mediate the dynamic control of poly(A) tail lengths of subsets of mRNAs are largely unclear, especially in mammals. Our work reveals an essential complex that controls the deadenylation of mRNAs during metaphase II arrest. It also provides a striking example of how the removal of a single protein can have disastrous consequences for the entire balance of protein synthesis and cell fate. Recently, *Btg4*^−/−^ mice have been reported to be infertile but otherwise viable [17]. This suggests that BTG4 is important primarily for reproduction, but not in other cells of the body. These properties make *Btg4* a good candidate whose mutation could contribute to the spontaneous abortive activation of eggs that has been implicated in infertility in humans [35,36]. Our findings reveal a major role of *Btg4* in the formation of a viable egg and suggest that the impaired embryonic development and female infertility observed in *Btg4^−/−^* mice [17] originate at least partially from defects during meiosis. It is conceivable that the imbalance in protein synthesis in *Btg4^−/−^* mutant mice also affects the stages after fertilization and thereby contributes to defects during embryo development. Interestingly, mutations in other BTG/TOB genes have also been linked to several types of cancer [37–40]. The mechanism of mRNA deadenylation by different BTG/TOB proteins is likely to be conserved. It is thus conceivable that the misregulation of BTG/TOB proteins can trigger a particularly efficient and dangerous route of cell reprogramming, which can have disastrous consequences not only for fertility but also in the context of cancer.

## 6. Material and methods

### 6.1. Preparation and culture of oocytes

All animals were maintained in a specific pathogen-free environment according to UK Home Office regulations. Fully grown oocytes from FVB females or follicle-enclosed oocytes from (C57BL × CBA) F_1_ females were isolated, microinjected and cultured as previously described [1]. All siRNAs were purchased from QIAGEN and 6 pl with a concentration of 1–6 µM were microinjected into follicle-enclosed oocytes:

BTG4

AAGGGCTTGTGCTGAGAGTAA

AAGCCTGAATATTTGACTCTA

AAGGAAGACTTGAAATAAATA

CNOT7

AGCAAGCATTCTGTTATGAAA

CAGGAAGTTGCTGAGCAGTTA

CTGGATGAAGAGATGAAGAAA

CNOT8

AAGGACAATTGTAAGAAGTAA

ATGCTTGTAAATATTATTTAT

TGCAATGTTGATCTTCTTAAA

TTGCTCTGAATTTGTAAACAA

EMI2

CGGCATGAACTTTAAAGGCAA

AAACTTCTAAATGATGATTTA

TCCATCAAATGTAATCTATAA

AAGCAGATCTCTGGAAAGTAA

MOS

CCCGAAGACTCCAACAGCCTA

AACAGGTATGTCTGATGCATA

CTCGGTGTATAAAGCCACTTA

CACGCCCAAAGCTGACATCTA

PABPN1

CCCAAAGGGTTTGCATATATA

CCGGAGCTAGAAGCGATCAAA

CCCAGTGATCATGTCTCTTGA

AGGAAGAGGCTGAGAAGCTAA

### 6.2. Expression constructs and mRNA synthesis

Previously published constructs were used for the visualization of chromosomes, pGEMHE-H2B–mRFP1 [41], or meiotic spindle, pGEMHE–EGFP–MAP4 [41] or pGEMHE–EGFP–α-tubulin [42]. The *Mus musculus Btg4* and *cyclin B* ORFs as well as *Emi2* and *cyclin A2* 3′UTR sequences were amplified from mouse oocyte cDNA. The *Btg4* ORF was inserted into pmEGFP-C1 and pmEGFP-N1 vectors (Clontech), using *Eco*RI and *Bam*HI. EGFP–Btg4 and Btg4–EGFP were then subcloned into pGEMHE, using *Age*I and *Bam*HI or *Nhe*I and *Not*I, respectively. pGEMHE–EGFP–Btg4 was mutated using the QuickChange II XL site-directed mutagenesis kit (Agilent). The *cyclin B* ORF was cloned into the pmCherry-N1 vector (Clontech), using *Age*I and *Nhe*I. Cyclin B-mCherry was then subcloned into the pGEMHE vector, using *Nhe*I and *Not*I. To obtain pGEMHE–securin–mCherry, the *Mus musculus* securin ORF was cloned into the pGEMHE vector, using *Eco*RI and *Bam*HI; and mCherry was sublconed into the same vector, using *Bam*HI and *Not*I. The *Mus musculus Cnot7* or *Cnot8* ORFs were ligated using the Gibson assembly kit (NEB) with pFlag-C3 that was linearized with *Xho*I. pT7-mEGFP-Emi2 3′UTR was created by ligating *EGFP* and the 3′ UTR of mouse *Emi2* by Gibson assembly and subcloning into a modified pmEGFP-N1 vector with added T7. For mRNA synthesis, the pGEMHE constructs were linearized with *Asc*I. pT7-mEGFP-Emi2 3′UTR was linearized with *Not*I. Capped mRNA was synthesized using T7 polymerase (mMessage mMachine kit, Ambion), following the manufacturer's instructions. Final mRNA concentrations were determined on ethidium bromide agarose gels by comparison of the total band intensity with an RNA standard (Ambion). When stated, mRNA tailing was performed using Poly(A) tailing kit (Ambion).

Capped mRNA was synthesized using T7 polymerase (mMessage mMachine kit, Ambion), following the manufacturer's instructions. Final mRNA concentrations were determined on ethidium bromide agarose gels by comparison of the total band intensity with an RNA standard (Ambion).

### 6.3. Detection of protein synthesis

MII or equivalent control and *Btg4*-depleted oocytes were incubated in M2 medium supplemented with 50 µM HPG for 2 h. The oocytes were fixed for 30 min at 37°C in 100 mM HEPES (pH 7; titrated with KOH), 50 mM EGTA (pH 7; titrated with KOH), 10 mM MgSO_4_, 2% formaldehyde (methanol free) and 0.2% Triton X-100, based on previously published methods. Oocytes were permeabilized in PBS with 0.5% Triton X-100 for 30 min at room temperature and washed in PBS + 3% BSA. HPG was detected, using Click-iT cell reaction kit (Life Technologies). The mean cytoplasmic signal was measured in the middle section of each oocyte.

### 6.4. Confocal microscopy

All images were acquired with a Zeiss LSM710 confocal microscope equipped with a Zeiss environmental incubator box or a Zeiss LSM780 confocal microscope equipped with a Tokai hit stage top incubator, with a C-Apochromat 40×/1.2 W water immersion objective lens. In most of the images, shot noise was reduced with a Gaussian filter, and maximum intensity z-projections are shown. The z-projections were generated in Zen (Zeiss).

### 6.5. Fluorescence measurements

The mean cytoplasmic signal of mCherry-tagged cyclin B or securin was measured in a selected area in the middle section of an oocyte over the entire course of meiosis, and the mean background signal from outside the boundaries of the oocyte was subtracted. The signal was then normalized to the starting intensity (relative fluorescence intensity equals 1). The measurements were aligned to anaphase onset in each oocyte (time 0). For EGFP measurements, nominal fluorescence values were compared and plotted over time from the start of the imaging.

### 6.6. Statistics

Mean, s.d. and statistical significance based on Student's *t*-test or Fisher's exact test (two-tailed) were calculated in Microsoft Excel. No statistical methods were used to predetermine sample size. All error bars show standard deviation. All box plots show median (line), mean (small square), 5th, 95th (whiskers) and 25th and 75th percentile (boxes).

### 6.7. Quantitative real-time PCR

mRNA was extracted from oocytes (10–30 per sample), using an RNeasy mini kit (Qiagen) for total RNA or Dynabeads mRNA DIRECT micro kit (Ambion) for the polyadenylated mRNA. The cDNA was generated, using a high-capacity RNA-to-cDNA kit (Applied Biosystems). Real-time PCR was performed with the 7900 HT real-time fast PCR system (Applied Biosystems), using SYBR Green as readout. GAPDH mRNA was used for normalization as the reference gene.

### 6.8. Transfection of HeLa cells and immunoprecipitation

HeLa cells were cultured in DMEM GlutaMAX medium (Gibco) at 37°C in 10% CO_2_. Cells were transfected using polyethylenimine. Proteins were immunoprecipitated using the magnetic GFP-Trap kit (ChromoTek) and dissociated from the beads by boiling in sample buffer. Immunoprecipitates were resolved on protein gels for western blot.

### 6.9. Yeast two-hybrid screen

The Y2H screen was performed by Hybrigenics, using full-length EGFP–BTG4 as a bait and a mouse ovarian cDNA library.

### 6.10. RNA sequencing

Total RNA was isolated, using NucleoSpin RNA XS (Macherey-Nagel) from 50 RNAi-treated oocytes at early metaphase II per sample. A cDNA library was prepared using SMARTer UltraLow Input RNA for sequencing (Clontech Laboratories), and the samples were processed by BGI Tech Solutions. The cDNA product was synthesized and amplified using a SMARTer PCR cDNA synthesis kit (Clontech Laboratories) from the total RNA (about 10 ng) of each sample. The cDNA was fragmented by Covaris E210, and the median insert length was about 200 base pairs. The paired-end cDNA library was prepared in accordance with Illumina's protocols with an insert size of 200 base pairs and sequenced for 100 base pairs by HiSeq4000 (Illumina). The differential gene expression was assessed using NOISeq and DESeq2, as described in the electronic supplementary material.

### 6.11. RNA-Seq analysis

RNA-Seq-based measurements of transcript abundances at the level of genes were represented by fragments per kilobase of transcript per million fragments mapped (FPKMs). FPKM is conceptually similar to the reads per kilobase per million reads sequenced (RPKM) measure, but it is easily adaptable for sequencing data from one to higher numbers of reads from single source molecules. To identify significantly differentially expressed genes between *Btg4*-depleted oocytes and the control group, we used a non-parametric method encoded in NOISeq and a parametric method DESeq2. For NOISeq, we first filtered for low count or abundance using the ‘CPM’ low count filter of NOISeq. Significant differential expression between groups was determined using NOISeq with the following parameters: (i) ‘tmm’, trimmed mean of log_2_ FPKM, normalization; (ii) biological replicates data; and (iii) probability of differential expression q being set to 0.8 or above and log_2_ values being greater than or equal to −1 or 1 for upregulated and downregulated genes, respectively, in *Btg4-*depleted oocytes. This yielded 149 upregulated and 12 downregulated genes in *Btg4*-depleted oocytes. Further, the DESeq2 standard pipeline for analyses was followed as described [43], which yielded 279 upregulated and 41 downregulated genes.

### 6.12. SDS–PAGE and immunoblotting

For western blot, 25–50 oocytes per sample were collected. Alternatively, cell lysates (input) or protein immunoprecipitates were collected as described above. Samples were heated at 95°C for 5 min with 1× sample buffer (NuPage LDS sample buffer, Invitrogen) with NuPage antioxidant (Invitrogen). Proteins were separated on 4–12% gradient NuPage bis–tris precast gels (Invitrogen) in MES–SDS running buffer (Formedium). Proteins were blotted onto nitrocellulose membranes (Amersham) at a constant current of 400 mA for 1 h. Membranes were blocked in 5% skimmed milk in PBST for 1 h at room temperature and incubated with primary antibodies (EMI2—Everest Biotech EB0606, Flag tag—Sigma F1804, p44/42 MAPK—Cell Signalling 91069, tubulin α—AbD Serotec MC178G) at 4°C overnight with secondary antibodies (Vector Laboratories PI-9500, GE Healthcare NA93 and NA934, Santa Cruz Biotechnology SC-2032) for 1–2 h at room temperature. The detection was performed, using ECL Prime or ECL Advanced (Amersham) and Kodak X-Ray films.

## Supplementary Material

Supplemental material

## Supplementary Material

Table S2 RNA-seq data

## Data accessibility

RNA sequencing data have been deposited in Gene Expression Omnibus (NCBI GEO) under accession number GSE83876.
