# Supplementary material for: The BTG4 and CAF1 complex prevents the spontaneous activation of eggs by deadenylating maternal mRNAs
Source: Open Biol. 2016 Sep 7;6(9):160184. doi: 10.1098/rsob.160184 (PMC5043581; doi:10.1098/rsob.160184)
Supplement: Supplemental material [file rsob160184supp1.pdf]

Supplemental material

Supplemental Figures

Figure S1

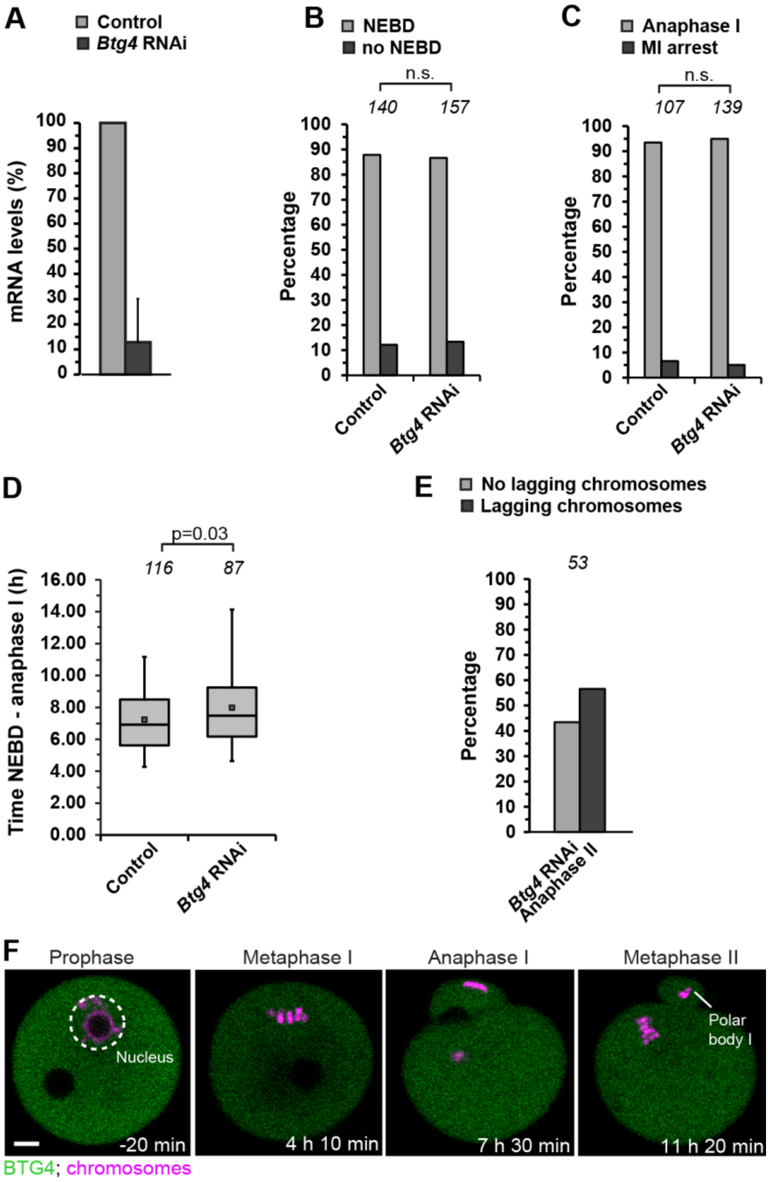

Figure S2

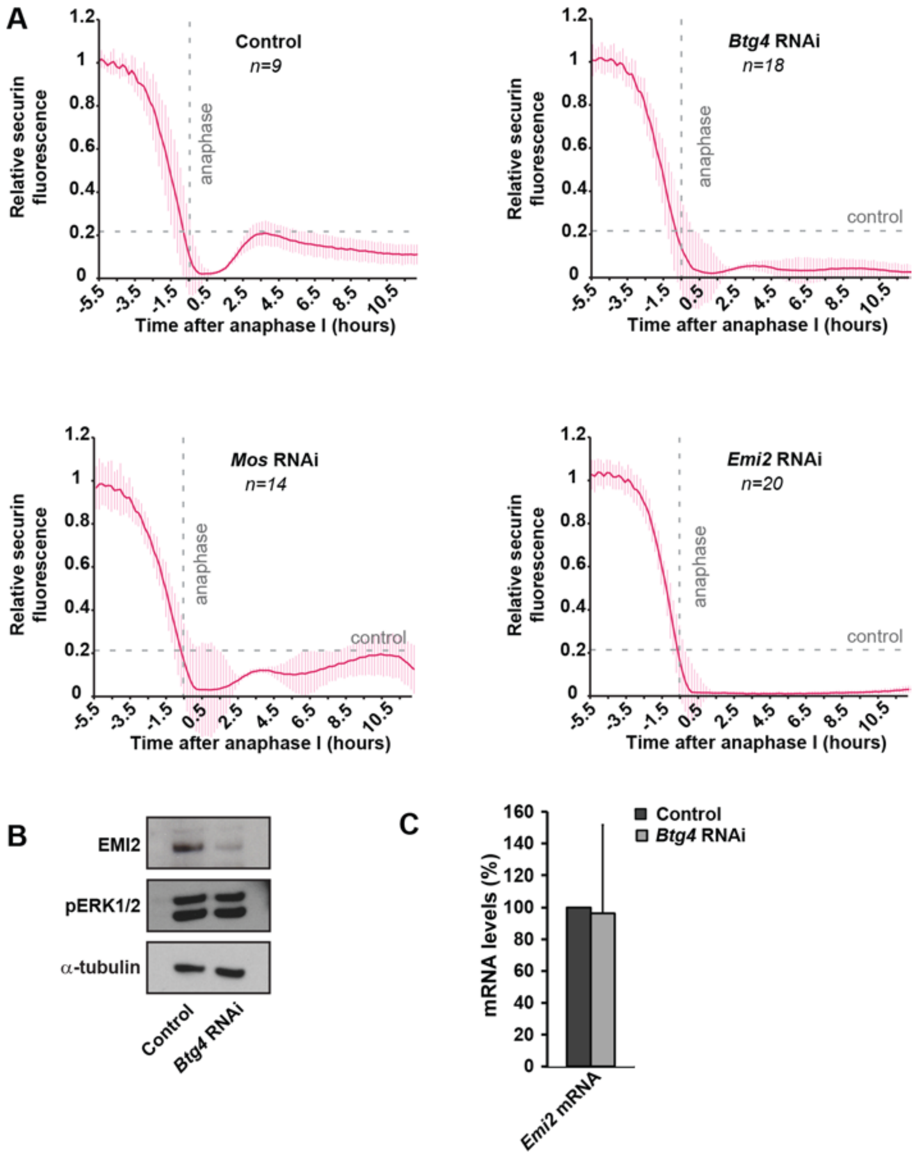
A
Control
n=9
Btg4 RNAi
n=18
Mos RNAi
n=14
Emi2 RNAi
n=20
Relative securin fluorescence
anaphase
control
Time after anaphase I (hours)
B
EMI2
pERK1/2
α-tubulin
Control
Btg4 RNAi
C
mRNA levels (%)
Control
Btg4 RNAi
Emi2 mRNA

Figure S3

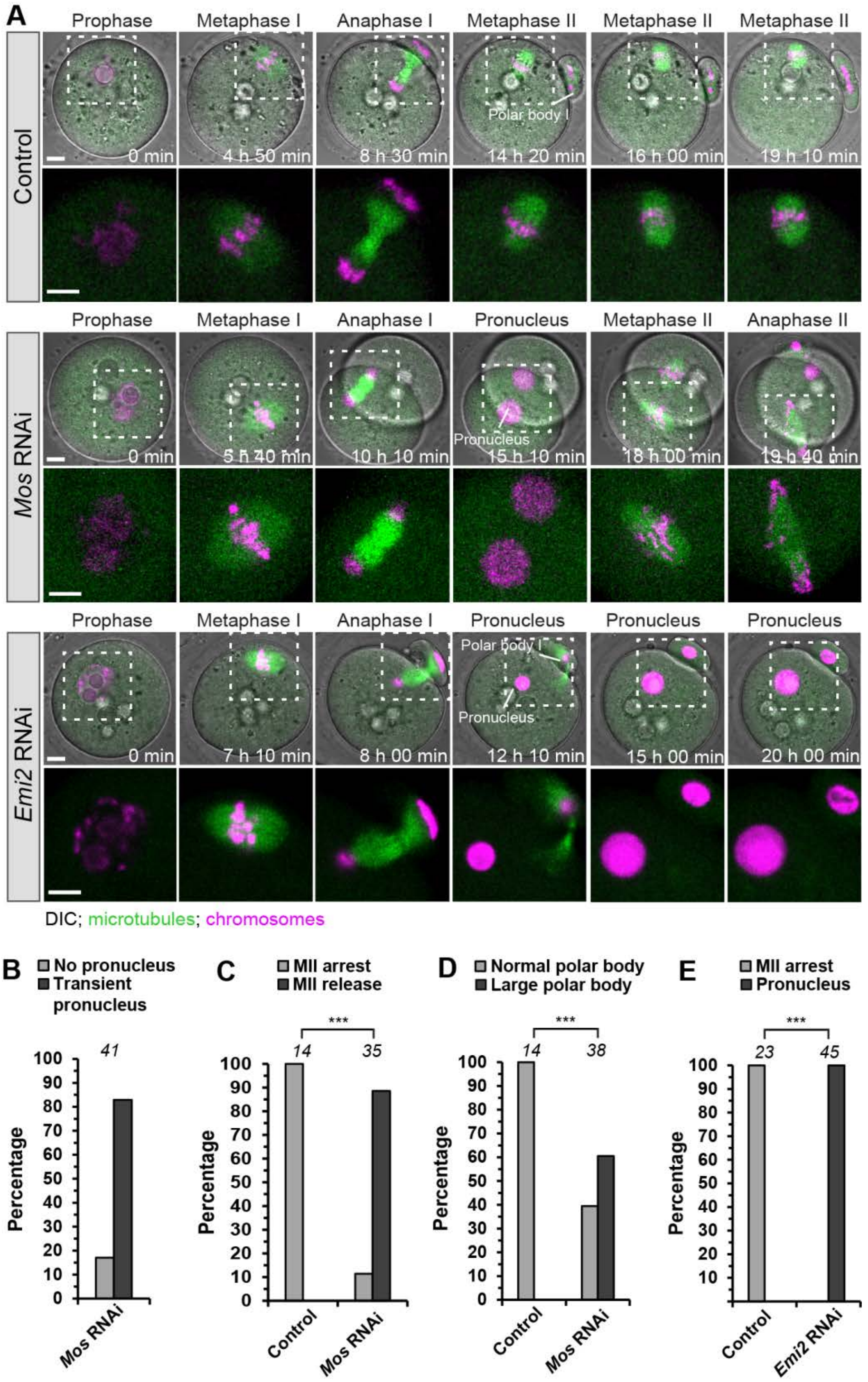
A
Prophase
Metaphase I
Anaphase I
Metaphase II
Metaphase II
Metaphase II
Control
Polar body I
0 min
4 h 50 min
8 h 30 min
14 h 20 min
16 h 00 min
19 h 10 min
Prophase
Metaphase I
Anaphase I
Pronucleus
Metaphase II
Anaphase II
Mos RNAi
Pronucleus
0 min
5 h 40 min
10 h 10 min
15 h 10 min
18 h 00 min
19 h 40 min
Prophase
Metaphase I
Anaphase I
Pronucleus
Pronucleus
Pronucleus
Emi2 RNAi
Polar body I
Pronucleus
0 min
7 h 10 min
8 h 00 min
12 h 10 min
15 h 00 min
20 h 00 min
DIC; microtubules; chromosomes
B
No pronucleus
Transient pronucleus
41
Percentage
Mos RNAi
C
MII arrest
MII release
***
14
35
Percentage
Control
Mos RNAi
D
Normal polar body
Large polar body
***
14
38
Percentage
Control
Mos RNAi
E
MII arrest
Pronucleus
***
23
45
Percentage
Control
Emi2 RNAi

Figure S4

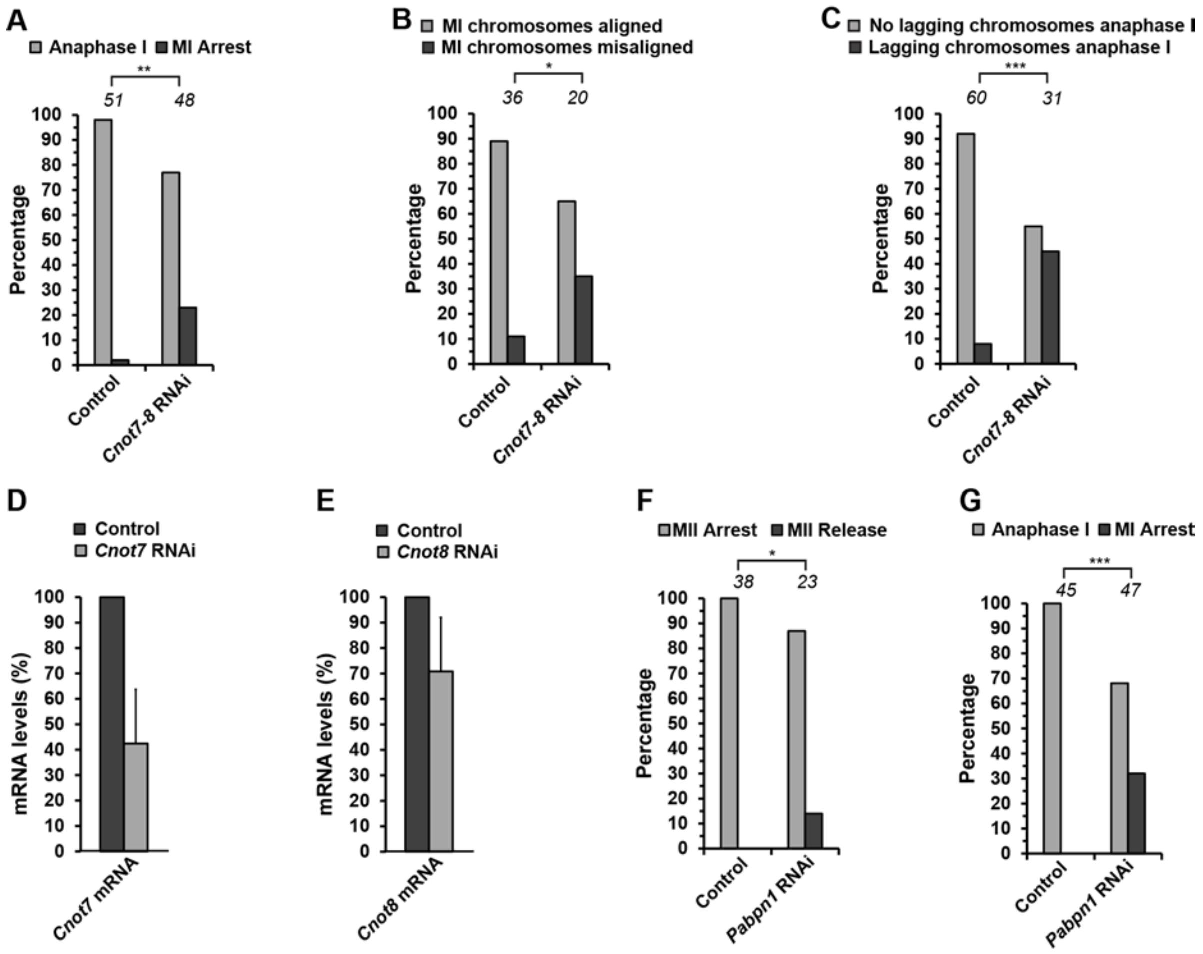
A
Anaphase I
MI Arrest
**
51
48
Percentage
Control
Cnot7-8 RNAi
B
MI chromosomes aligned
MI chromosomes misaligned
*
36
20
Percentage
Control
Cnot7-8 RNAi
C
No lagging chromosomes anaphase I
Lagging chromosomes anaphase I
***
60
31
Percentage
Control
Cnot7-8 RNAi
D
Control
Cnot7 RNAi
mRNA levels (%)
Cnot7 mRNA
E
Control
Cnot8 RNAi
mRNA levels (%)
Cnot8 mRNA
F
MII Arrest
MII Release
*
38
23
Percentage
Control
Pabpn1 RNAi
G
Anaphase I
MI Arrest
***
45
47
Percentage
Control
Pabpn1 RNAi

Figure S5

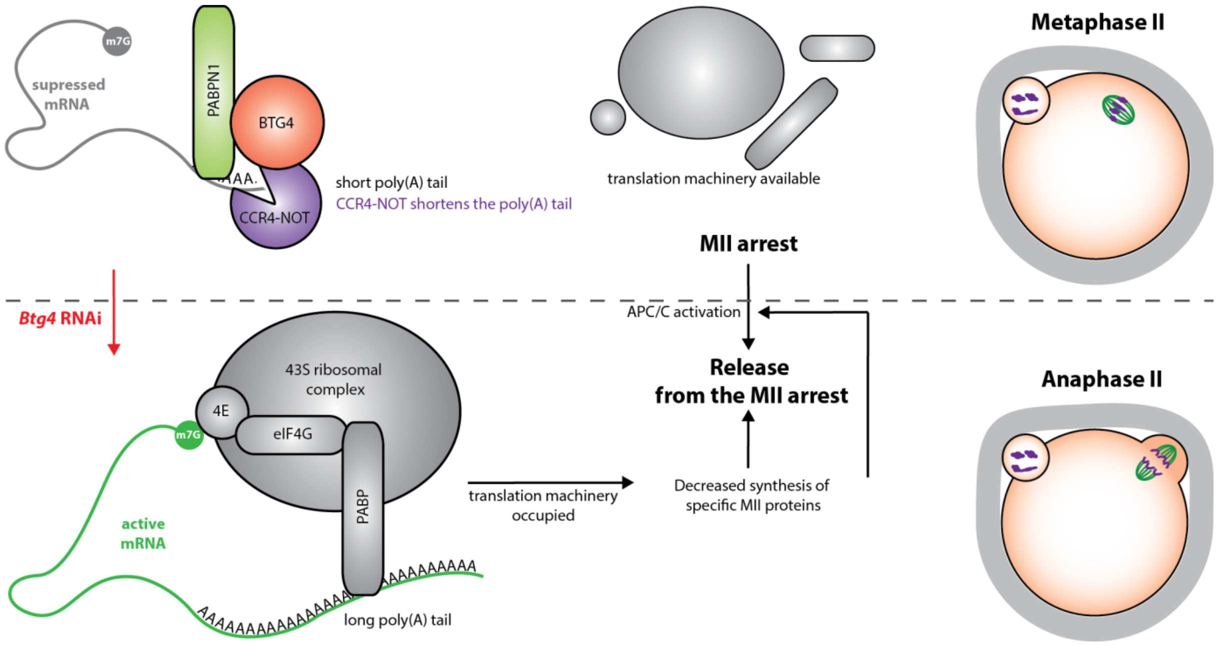

Supplemental Figure Legends

Figure S1: Evaluation of the phenotype following *Btg4* depletion

(A) The efficiency of the RNAi knockdown as quantified by Real Time qPCR. **(B-D)** Meiotic progression of Btg4-depleted oocytes, showing that the efficiency of the NEBD (B) and anaphase I onset (C) was not affected and the timing of anaphase I onset was similar to the control oocytes (D). **(E)** Chromosome lagging during anaphase II in *Btg4*-depleted oocytes. **(F)** Cytoplasmic and nuclear localization of exogenously expressed EGFP-BTG4 during oocyte maturation. Single z sections are shown. Time stamp denotes time after the NEBD. The number of analyzed oocytes is specified above the bars. *P* values were calculated with Fisher's exact test (B,C) or Student t-test (D) and n.s. denotes no statistical significance (*p*-value > 0.05). Data from 4 independent experiments for (A), 13 for (B) and (C), 10 for (D) and 11experiments for (E).

Figure S2: *Btg4*-depleted oocytes fail to inhibit the APC/C and have reduced levels of EMI2

(A) Quantifications of securin signal intensity measured in the middle section of each oocyte, reflecting changes in the APC/C activity over time (time 0 was set as the anaphase onset). High fluorescence correlates with low

APC/C activity. Vertical dashed line indicates anaphase onset and the horizontal dashed line indicates the fluorescence recovery in control oocytes during metaphase II. **(B)** *Btg4*-depleted oocytes have decreased levels of EMI2 protein while the activation of MAPK (pERK1/2) was not affected as indicated by phosphorylated Thr202/Tyr204 on ERK1 and Thr185/Tyr187 on ERK2. **(C)** The levels of polyadenylated *Emi2* mRNA were not changed in *Btg4*-depleted oocytes at metaphase II. Data from 2 independent repetitions in (A), 5 in (B) and 3 in (C).

Figure S3: Depletion of *Mos* triggers spontaneous resumption of meiosis while depletion of *Emi2* results in meiotic exit

(A) Representative examples of oocytes microinjected with siRNAs targeting *Btg4, Mos* or *Emi2* or microinjected with control siRNA. Region of spindle and chromosomes, highlighted in the upper panel, is magnified without DIC below. Scale bar shows 10 µm. Time stamp denotes time after the start of imaging. (**B)** 83% of *Mos*-depleted oocytes transiently form a pronucleus after progressing through anaphase I and before forming metaphase II spindle. (**C)** 89% of *Mos*-depleted oocytes that formed metaphase II spindle spontaneously resumed meiosis and progressed into anaphase II, **(D)** *Mos*-depleted oocytes extruded unusually large 1st polar bodies. **(E)** *Emi2*-depleted oocytes exited meiosis and formed pronuclei after the completion of anaphase I. The number of analyzed oocytes is specified above the bars. *P* values were calculated with Fisher's exact test and *** denotes *p*-value < 0.0001. Data from 4 experiments for (B-D) and 5 for (E).

Figure S4: Defects observed upon depletion of CAF1 (*Cnot7* and *Cnot8*) and *Pabpn1*

Co-depletion of *Cnot7* and *Cnot8* resulted in a high incidence of metaphase I arrest **(A)**, chromosome misalignment during metaphase I **(B)** and lagging chromosomes during anaphase I **(C)**. **(D-E)** RNAi efficiency of *Cnot7* and *Cnot8* knockdown, respectively, as quantified by Real Time qPCR. **(F-G)** Depletion of *Pabpn1* resulted in the spontaneous resumption of meiosis (F) and a high incidence of metaphase I arrest (G). The number of analysed oocytes is specified above the bars. *P* values were calculated with Fisher's exact test and *** denotes a *p*-value < 0.0001, ** denotes a *p*-value < 0.01 and * denotes a *p*-value < 0.05. Data from 6 experiments for (A-C), 3 for (D), 2 for (E) and 5 for (F-G).

Figure S5: Model for the function of BTG4 in metaphase II arrest

Supplemental Movie Legends

Movie S1

Live oocytes microinjected with control siRNA (Control) or siRNAs targeting *Btg4* (Btg4 RNAi) expressing mEGFP-MAP4 (green, microtubules) and H2B-mRFP (magenta, chromosomes). Scale bar shows 10 µm.

Movie S2

Live oocytes microinjected with control siRNA (Control) or siRNAs targeting *Cnot7 and Cnot8* (Cnot7-8 RNAi) expressing mEGFP-MAP4 (green, microtubules) and H2B-mRFP (magenta, chromosomes). Scale bar shows 10 µm.

Movie S3
Live metaphase II oocytes microinjected with control mRNA encoding mEGFP (EGFP) or polyadenylated mRNA encoding mEGFP (EGFP-polyA), expressing H2B-mRFP (magenta, chromosomes). Control group expressing only H2B-mRFP. Scale bar shows 10 µm.

Table S1Results of the Y2H screen

Results Summary

ULTImate Y2H SCREEN
Mus musculus - Btg4
# vs Mouse Ovaries_RP1

Tue, Aug 12, 2014 - 10:43 AM

Screen Parameters

Nature	cDNA
Reference Bait Fragment	Mus musculus - Btg4 (aa 1-250) ; hgx3704v1
Prey Library	Mouse Ovaries_RP1
Vector(s)	pB27 (N-LexA-bait-C fusion) + pB66 (N-GAL4-bait-C fusion)
Processed Clones	24 (pB27_A) + 38 (pB66_A) + 28 (pB66_B)
Analyzed Interactions	140 millions (pB27_A) + 41.6 millions (pB66_A) + 16.8 millions (pB66_B)
3AT Concentration	0.0 mM (pB27_A) + 0.0 mM (pB66_A) + 0.0 mM (pB66_B)

Global PBS®

Global PBS (for Interactions represented in the Screen)		Nb	%
A	Very high confidence in the interaction	0	0.0%
B	High confidence in the interaction	2	9.1%
C	Good confidence in the interaction	3	13.6%
D	Moderate confidence in the interaction<br>This category is the most difficult to interpret because it mixes two classes of interactions :<br>- False-positive interactions<br>- Interactions hardly detectable by the Y2H technique (low representation of the mRNA in the library, prey folding, prey toxicity in yeast)	15	68.2%
E	Interactions involving highly connected (or relatively highly connected) prey domains, warning of non-specific interaction. The total number of screens performed on each organism is taken into account to set this connectivity threshold: 20 interactions to different bait proteins in our entire database for Human, 10 for Mouse, Drosophila and Arabidopsis and 6 for all other organisms. They can be classified in different categories:<br>- Prey proteins that are known to be highly connected due to their biological function<br>- Proteins with a prey interacting domain that contains a known protein interaction motif or a biochemically promiscuous motif	1	4.5%
F	Experimentally proven technical artifacts	1	4.5%
Non Appliable			
N/A	The PBS is a score that is automatically computed through algorithms and cannot be attributed for the following reasons :<br>- All the fragments of the same reference CDS are antisense<br>- The 5p sequence is missing<br>- All the fragments of the same reference CDS are either all OOF1 or all OOF2<br>- All the fragments of the same reference CDS lie in the 5' or 3' UTR		

HYBRIGENICS CONFIDENTIAL - 932067 - M. Pasternak

HYBRIGENICS SERVICES

Prey Fragment Analysis

Symbols	Means
	The fragment contains the full length CDS
	Fragment is fully in 5' UTR
	Fragment is fully in 3' UTR
✕	Fragment contains at least one In Frame STOP codon
[NR]	Fragment was found to be non relevant (poor quality, high N density)
IF<br>OOF1<br>OOF2	With regard to the theoretical frame of each corresponding CDS (GeneBank),fragments are cloned in frame (IF) if they are in the same frame as Gal4AD.In general, polypeptides synthesized from OOF fragments are not considered of biological interest, unless found together with another frame. However, some of the proteins expressed from an OOF fragment can be translated in the correct frame,due to the existence of natural frame-shift events during translation in yeast
??	Unidentified frame when :<br>- The clone sequence is antisense<br>- The 5p sequence is missing
N	Antisense
Start...Stop	Position of the 5p and 3p prey fragment ends, relative to the position of the ATG start codon (A=0)

Clone Name	Type Seq	Gene Name (Best Match)	Start..Stop (nt)	Frame	Sense	%Id 5p	%Id 3p	PBS
pB66_B-6	5p/3p	Mus musculus - Ahcyl2	384..1465	IF		98.1	97.9	D
pB27_A-10	5p/3p	Mus musculus - Anpep	2667..3096 ✕	IF		98.1	97.4	D
pB27_A-18	5p/3p	Mus musculus - Cnot7	-287..941 ✕	OOF2		97.4	98.7	B
pB66_B-10	5p/3p	Mus musculus - Cnot7	-287..941 ✕	OOF2		98.2	96.3	B
pB66_A-29	3p	Mus musculus - Cnot7	..-264	??	N		88.7	N/A
pB66_A-21	5p/3p	Mus musculus - Cnot7	16..927 ✕	OOF1		95.8	95.6	B
pB66_B-26	5p/3p	Mus musculus - Cnot7	39..934 ✕	IF		97.0	94.1	B
pB27_A-20	5p/3p	Mus musculus - Cnot7	920..-213	??	N	99.3	98.0	N/A
pB66_B-2	5p/3p	Mus musculus - Cnot7	920..-262	??	N	97.6	97.7	N/A
pB66_A-12	5p/3p	Mus musculus - Cnot7	920..-262	??	N	96.1	95.4	N/A
pB27_A-24	5p/3p	Mus musculus - Cnot7	927..-260	??	N	99.3	99.4	N/A
pB27_A-19	5p/3p	Mus musculus - Cnot7	927..-269	??	N	98.8	95.9	N/A
pB66_B-15	5p/3p	Mus musculus - Cnot7	927..-260	??	N	97.5	96.9	N/A
pB66_A-15	5p/3p	Mus musculus - Cnot7	927..-260	??	N	96.4	95.7	N/A
pB66_A-31	5p/3p	Mus musculus - Cnot7	927..-231	??	N	95.0	93.1	N/A
pB66_A-13	5p/3p	Mus musculus - Cnot7	928..-219	??	N	97.6	95.7	N/A
pB27_A-16	5p/3p	Mus musculus - Cnot7	939..-263	??	N	99.3	98.2	N/A
pB27_A-11	5p/3p	Mus musculus - Cnot7	939..-219	??	N	98.9	98.3	N/A
pB27_A-2	5p/3p	Mus musculus - Cnot7	939..-253	??	N	97.1	94.8	N/A
pB27_A-4	5p/3p	Mus musculus - Cnot7	939..-263	??	N	97.2	94.7	N/A
pB66_A-3	5p/3p	Mus musculus - Cnot7	939..-219	??	N	98.0	95.6	N/A
pB66_A-28	5p/3p	Mus musculus - Cnot7	939..-263	??	N	93.5	89.1	N/A
pB66_A-30	5p/3p	Mus musculus - Cnot7	939..-234	??	N	97.7	95.5	N/A
pB66_B-30	5p/3p	Mus musculus - Cnot7	939..-253	??	N	98.0	96.2	N/A
pB27_A-1	5p/3p	Mus musculus - Cnot7	944..-257	??	N	99.5	99.1	N/A
pB27_A-13	5p/3p	Mus musculus - Cnot7	944..-257	??	N	95.7	98.1	N/A
pB27_A-6	5p/3p	Mus musculus - Cnot7	944..-257	??	N	99.3	98.1	N/A
pB27_A-22	5p/3p	Mus musculus - Cnot7	944..-276	??	N	99.1	99.0	N/A
pB66_B-8	5p/3p	Mus musculus - Cnot7	944..-257	??	N	97.9	96.9	N/A

HYBRIGENICS CONFIDENTIAL - 932067 - M. Pasternak

Clone Name	Type Seq	Gene Name (Best Match)	Start..Stop (nt)	Frame	Sense	%Id 5p	%Id 3p	PBS
pB66_A-10	5p/3p	Mus musculus - Cnot7	944..-257	??	N	98.6	97.5	N/A
pB66_B-11	5p/3p	Mus musculus - Cnot7	944..-276	??	N	96.9	95.2	N/A
pB66_B-12	5p/3p	Mus musculus - Cnot7	944..-257	??	N	98.4	97.5	N/A
pB66_A-16	5p/3p	Mus musculus - Cnot7	944..-276	??	N	94.5	92.9	N/A
pB66_B-25	5p/3p	Mus musculus - Cnot7	944..-257	??	N	94.9	96.0	N/A
pB66_B-35	5p/3p	Mus musculus - Cnot7	944..-276	??	N	96.9	95.6	N/A
pB66_B-27	5p/3p	Mus musculus - Cnot7	944..-257	??	N	97.8	96.7	N/A
pB66_B-3	5p/3p	Mus musculus - Cnot7	947..-197	??	N	97.9	93.6	N/A
pB66_A-23	5p/3p	Mus musculus - Cnot7	947..-197	??	N	97.8	95.9	N/A
pB66_B-36	5p/3p	Mus musculus - Cnot7	947..-197	??	N	97.4	95.5	N/A
pB66_A-5	5p/3p	Mus musculus - Cnot7	949..-188	??	N	96.4	93.6	N/A
pB66_A-6	5p/3p	Mus musculus - Cnot7	949..-188	??	N	93.0	89.2	N/A
pB66_B-29	5p/3p	Mus musculus - Cnot7	949..-188	??	N	97.7	96.5	N/A
pB66_A-20	5p/3p	Mus musculus - Cnot7	1157..-262	??	N	94.4	92.2	N/A
pB66_A-25	5p/3p	Mus musculus - Cnot7	1157..-262	??	N	96.1	94.9	N/A
pB66_B-1	5p/3p	Mus musculus - Cnot7	1168..-287	??	N	94.8	92.7	N/A
pB66_A-7	5p/3p	Mus musculus - Cnot7	1301..-263	??	N	93.8	90.7	N/A
pB66_A-9	5p/3p	Mus musculus - Cnot7	1301..-264	??	N	92.7	89.8	N/A
pB66_B-5	5p/3p	Mus musculus - Cnot7	1301..-263	??	N	94.1	93.0	N/A
pB27_A-21	5p/3p	Mus musculus - Cnot7	1301..-264	??	N	99.5	93.5	N/A
pB27_A-9	5p/3p	Mus musculus - Cnot7	1437..-219	??	N	98.9	97.9	N/A
pB27_A-8	5p/3p	Mus musculus - Cnot7	1437..-219	??	N	99.1	98.3	N/A
pB27_A-17	5p/3p	Mus musculus - Cnot7	1437..-219	??	N	97.8	97.7	N/A
pB66_B-4	5p/3p	Mus musculus - Cnot7	1437..-219	??	N	96.2	93.4	N/A
pB66_B-32	5p/3p	Mus musculus - Cnot7	1437..-219	??	N	96.9	93.6	N/A
pB27_A-23	5p/3p	Mus musculus - Cnot8	-7..1020	IF		99.6	98.7	D
pB27_A-7	5p/3p	Mus musculus - Cnot8	-7..1020	IF		95.9	98.3	D
pB66_B-24	5p/3p	Mus musculus - Cnot8	-7..1020	IF		96.6	93.8	D
pB66_A-24	5p	Mus musculus - Col6a1	1152	IF		97.9		C
pB66_A-27	5p/3p	Mus musculus - Col6a1	1188..2027	IF		95.7	92.1	C
pB66_A-32	3p	Mus musculus - Dnajc7	..757	??			95.0	E
pB66_B-18	5p/3p	Mus musculus - Dnajc7	12..1032	IF		90.7	88.3	E
pB66_A-40	5p/3p	Mus musculus - Dnajc7	18..1035	IF		96.2	95.4	E
pB66_A-19	3p	Mus musculus - Efhc1	615..1056	IF			64.7	D
pB66_B-19	5p/3p	Mus musculus - Gemin4	1443..3209	IF		96.6	93.5	D
pB66_B-9	5p/3p	Mus musculus - Gemin4	1443..3209	IF		98.6	95.2	D
pB27_A-15	5p/3p	Mus musculus - Hey2	-94..430	IF		100.0	99.8	D
pB27_A-5	5p/3p	Mus musculus - Heyl	-4..1669	IF		100.0	98.7	F
pB27_A-3	5p/3p	Mus musculus - Heyl	-4..1669	IF		97.2	95.7	F
pB66_A-34	5p/3p	Mus musculus - Hook3	1278..2108	IF		97.4	96.0	C
pB66_B-39	5p/3p	Mus musculus - Hook3	1506..2942	IF		97.6	97.1	C
pB66_A-14	5p/3p	Mus musculus - Mdfic	126..795	IF		99.7	98.2	B
pB66_A-11	5p/3p	Mus musculus - Mdfic	219..795	IF		100.0	100.0	B
pB66_A-39	5p/3p	Mus musculus - Mdfic	243..693	IF		99.8	100.0	B
pB66_A-35	5p/3p	Mus musculus - PAP homologousprotein	219	IF		90.2	60.8	D
pB66_B-23	5p/3p	Mus musculus - Pabpn1	492..882	IF		100.0	100.0	C
pB66_A-37	5p/3p	Mus musculus - Pabpn1	495..1746	IF		93.9	94.2	C
pB66_A-38	5p/3p	Mus musculus - Plcl2	4116..3495	??	N	100.0	100.0	N/A
pB66_B-21	5p/3p	Mus musculus - Pole2	-7..728	IF		94.0	95.9	D
pB66_A-33	3p	Mus musculus - Stag2	..2163	??			87.1	N/A

Clone Name	Type Seq	Gene Name (Best Match)	Start..Stop (nt)	Frame	Sense	%Id 5p	%Id 3p	PBS
pB66_A-17	5p/3p	Mus musculus - Syne1	4524..5734	IF		95.0	93.1	D
pB66_A-4	5p/3p	Mus musculus - Syne1	4524..5734	IF		96.9	94.8	D
pB66_A-2	5p/3p	Mus musculus - Syne1	4524..5734	IF		94.3	93.0	D
pB66_B-34	5p/3p	Mus musculus - Syne1	4524..5734	IF		97.7	95.0	D
pB66_A-26	5p/3p	Mus musculus - Syne1	4524..5734	IF		96.5	93.9	D
pB66_A-36	3p	Mus musculus - GenMatch GI:482677759	-1..148 ✗	IF			100.0	D
pB27_A-14	5p/3p	Mus musculus - GenMatch GI:77404123	-2..132	IF		100.0	100.0	D
pB66_A-22	5p/3p	Mus musculus - GenMatch GI:60543593	-1..539 ✗	IF		97.0	98.5	D
pB66_A-18	5p/3p	Mus musculus - GenMatch GI:77404128	-1..680 ✗	IF		98.8	99.4	D
pB27_A-25	5p/3p	Mus musculus - GenMatch GI:24462435	-1..754 ✗	IF		99.5	98.3	D
pB66_B-17	5p/3p	Mus musculus - GenMatch GI:170932460	-1..943 ✗	IF		100.0	72.7	D

HYBRIGENICS CONFIDENTIAL - 932067 - M. Pasternak
